# Traction, Contraction … Attraction

**Published:** 2008-02-15

**Authors:** Dimitrios Karypidis

**Affiliations:** Plastic Surgery Department, ‘A. Sygros’ Hospital of Dermatologic Disease, Athens, Greece; and Wound Healing Research Unit, Cardiff University, Cardiff, United Kingdom

Historically, at first it was believed that contraction forces were directly applied by the newly synthesized collagen at the site of the wound. Later it was shown that cells mediate this process. Fibroblasts are considered to have the capital role of mediating contraction that appears to be a coordinated multistage process in both the intracellular and extracellular levels. The fibrous and structural matrix proteins that comprise the interconnecting scaffold in between wounded tissue and the fibroblasts undergoing functional modification toward facilitating a process of decreasing the physical distance of the wound parts so as to cover spatial defects remain rather undisputed. Lately, theories reflect a dual perspective regarding cellular mechanisms. According to the first, differentiated myofibroblasts^1^ function collectively as a relatively consistent mechanism of muscle-like contraction by actively inducing their contractile properties as a whole. Alternatively, fibroblasts do not seem to utilize any contractile capacity as a whole but exhibit an accumulative effect of decreasing interstitial distances by individually exerting traction forces.^2^,^3^

One important area is the signaling of fibroblasts differentiation-activation cascade. Fibronectin has been shown to play an important role in regulating the migration and the subsequent proliferation of fibroblasts, interacting by both integrin and nonintegrin receptors and controlling the process of mechanotransduction via changes in its stereochemical structure. More specifically the extent to which the initial forms of fibronectin are “unfolded” serves both as a matrix-originating signal to the cells and as a main compartment of their subsequent environment of migration and organization.^4^ In addition, the type of collagen (type III collagen) has been demonstrated to be a determining factor of the speed, the intensity, and the quality of contraction.

Alternatively, recently shown facts support the suggestion that cytokinesis and contraction are a process controlled by intracellular factors. Cytoskeleton microtubule consistency and focal organization of F-actin and myosin II toward a dynamic process of initiating contraction has been shown to be regulated by small intracellular signaling factors such as the Rho GTPases.^5^

In addition to the identified role of integrin family, cell adhesion molecules, selectins, and cadherins, recent research on the role of membrane receptors offers increasing support to the role of gap junction intercellular communication between fibroblasts.^6^

Extracellular cytokines have also been scrutinized. Results concern mainly growth factors that present both direct and indirect effects on wound contraction.^7^ Transforming growth factor β (TGF-β) seems to orchestrate the process, namely but not solely by stimulating fibroblasts differentiation, migration, and organization. Other examples include platelet derived growth factor that stimulates the production of TGF-β and fibroblast growth factor 2 (basic), which is a chemotactic and mitogenic factor. Recently, neurotrophin nerve growth factor was shown to increase the contraction of differentiated fibroblasts in collagen gel, implying a potential role in wound contraction and repair, nevertheless, in need of further investigation.^8^ Regarding the role of growth factors, it would be more useful to consider them as mediators of a very balanced cell–cell and cell–extra cellular matrix (ECM) interaction rather than as omnipotent modulators.^9^

Conclusively, recent evidence derives from a rapidly increasing range of research, such as the genetic regulation of fibroblast phenotypic modification and the diverse and multifactorial molecular properties of the ECM, providing a more efficient and systematic approach of the wound contraction phenomenon.
